# Autophagy-related biomarkers in hepatocellular carcinoma and their relationship with immune infiltration

**DOI:** 10.1007/s12672-024-01167-x

**Published:** 2024-07-23

**Authors:** Tingting Li, Lin Zhang

**Affiliations:** Clinical Laboratory Department, the Fifth Affiliated Hospital of Zhengzhou University, Zhengzhou, China

**Keywords:** Autophagy, Hepatocellular carcinoma, Information biology, Immune infiltration, Prognosis

## Abstract

**Background:**

Autophagy regulation plays vital roles in many cancers. We aimed to investigate the expression, prognostic value, and immune infiltration of autophagy-related genes in hepatocellular carcinoma (HCC) by bioinformatics analysis.

**Method:**

Human autophagy-related differentially expressed genes (DEGs) between adjacent and HCC tissues were identified. We performed Gene Ontology (GO) and Kyoto Encyclopedia of Genes and Genomes (KEGG) enrichment analyses. We also evaluated immune infiltration and the response to tumor-sensitive drugs. Finally, we verified the expression of these proteins in clinical samples by immunohistochemistry (IHC), RNA isolation and real-time reverse transcription polymerase chain reaction (RT‒PCR).

**Results:**

A total of 57 autophagy-related DEGs were identified. The HUB genes (*BIRC5, CDKN2A, SPP1,* and *IGF1*) were related to the diagnosis and prognosis of HCC. The HUB genes were significantly enriched in immune-related pathways. Furthermore, correlation analysis revealed that HUB gene expression was associated with immune infiltration. We identified 35 tumor-sensitive drugs targeting the HUB genes. Finally, by IHC, we discovered that the protein of CDKN2A, BIRC5, and SPP1 were upregulated in HCC tissues, while IGF1 was downregulated in HCC tissues compared with the levels in paracarcinoma tissues; by RT‒PCR, we discovered that the mRNA of *CDKN2A, BIRC5,* and *SPP1* were upregulated in HCC tissues, while the mRNA of *IGF1* was downregulated in HCC tissues compared with the levels in paracarcinoma tissues.

**Conclusion:**

We screened and validated four autophagy-related genes associated with immune infiltration and prognosis in patients with HCC.

## Introduction

Hepatocellular carcinoma (HCC) is a prevalent malignancy and ranks third in cancer-related mortality worldwide [1]. Chronic infection with hepatitis C virus (HCV) or hepatitis B virus (HBV) is the leading cause of HCC [2]. Among primary liver cancers, HCC is the most common type [3]. Considering the harmfulness and complexity of HCC, identifying potential molecular biomarkers and therapeutic targets is vital for the early diagnosis of HCC.

Autophagy is a lysosome-dependent pathway that maintains cellular homeostasis and controls cellular components by promoting the clearance of misfolded proteins and damaged organelles [4]. Autophagy plays dual roles in HCC, as it not only inhibits the formation of malignant tumors but also contributes to the persistence of cancer [5]. Evidence suggests that autophagy is critical for tumor growth, metastasis, and therapeutic resistance [6]. For example, autophagy inhibits carcinogenesis through the mTOR and AMPK pathways, and autophagy promotes carcinogenesis through the mTOR, class I PI3K, and AKT pathways [7].

The present study aimed to elucidate the link between autophagy-related genes and HCC prognosis. We screened and validated the autophagy-related genes predicted to be involved in HCC, and we explored the roles of these genes in immune infiltration.

## Materials and methods

### Collection of GEO and TCGA datasets and autophagy-related genes

We collected the gene expression dataset GSE112790 from the GEO database (https://www.ncbi.nlm.nih.gov/). The GSE112790 dataset contains 15 adjacent liver tissue samples and 183 hepatocellular carcinoma samples. Standardized RNA-Seq data were collected from the TCGA database (https://portal.gdc.cancer.gov). A total of 796 human autophagy-related genes were downloaded from HAMdb (http://hamdb.scbdd.com/).

### Identification of DEGs

The differentially expressed genes (DEGs) were identified between adjacent noncancerous tissues and hepatocellular carcinoma tissues based on a |logFC|> 1 and an adjusted p < 0.05. A volcano map was created with an online bioinformatics tool (http://www.bioinformatics.com.cn/). The intersections of up- or downregulated autophagy-related genes were obtained using the Venn tool.

### Functional enrichment analysis of autophagy-related genes

Gene Ontology (GO) and Kyoto Encyclopedia of Genes and Genomes (KEGG) enrichment analyses were performed with the R package clusterProfiler (4.4.4). A p < 0.05 was considered to indicate significant enrichment. The visual bubble plots for the GO-BP and KEGG enrichment analyses were generated with the R package ggplot2 (3.3.6).

### Protein‒protein interaction (PPI) network construction and hub gene identification

The String database (https://string-db.org/) was used to construct a PPI network for autophagy-related genes with a minimum required score of 0.4. Hub genes were further characterized by cytoHubba.

### Human Protein Atlas (HPA)

The HPA database is an open database that allows researchers to explore the human proteome. In this study, we used the HPA database (http://www.proteinatlas.org/) to verify the protein expression of four hub genes selected from adjacent noncancerous tissues and hepatocellular carcinoma tissues by immunohistochemistry.

### Verification of the mRNA expression of IGF1, CDKN2A, BIRC5 and SPP1

The differences in the mRNA expression levels of *IGF1, CDKN2A, BIRC5,* and *SPP1* between tumor and nontumor tissues were verified from the GSE84402 dataset, in which 28 samples were included, consisting of 14 hepatocellular carcinoma samples and 14 nontumor samples.

The TCGA database was also used to validate the expressions of *IGF1, CDKN2A, BIRC5,* and *SPP1* in HCC. There were 424 samples, including 374 tumor tissues and 50 tumor-adjacent tissues. Statistical significance was indicated by p < 0.05.

### Survival analysis, Receiver Operating Characteristic (ROC) curves, and Tumor Node Metastasis (TNM) Stages of IGF1, CDKN2A, BIRC5, and SPP1 for HCC

Survival analysis was performed in R, and the results were visualized using the survminer and ggplot2 packages.

ROC curve analysis was performed, and areas under the ROC curve were calculated using the R packages pROC and ggplot2.

TNM stages were determined by using the R packages ggplot2 (3.3.6), stats (4.2.1), and car (3.1.0).

### Gene enrichment analysis

Standardized RNA-Seq data collected from the TCGA database were used for gene set enrichment analysis (GSEA). This study analyzed GO and GSEA data to investigate the possible biological functions of IGF1, CDKN2A, BIRC5, and SPP1 in HCC. A P.adj < 0.05 and a false discovery rate (FDR) < 0.25 were considered to indicate statistical significance.

### Tumor immune infiltration analysis

The TIMER database is an integrated resource for systematically analyzing the abundance of immune infiltrates in diverse cancer types (https://cistrome.shinyapps.io/timer/). In this study, the TIMER database was used to explore the correlations between the infiltration of IGF1, CDKN2A, BIRC5, and SPP1 and immune cells (dendritic cells, neutrophils, macrophages, CD4 + T cells, CD8 + T cells, and B cells).

Twenty-four immune cell infiltrates and their correlations with the levels of BIRC5, CDKN2A, SPP1, and IGF1 were analyzed by the R package ggplot2 (3.3.6).

Finally, we evaluated the correlations of the immune checkpoints with the HUB genes on the XIANTAO platform (https://www.xiantaozi.com/).

### Analysis of the sensitivity to potential therapeutic drugs

Using the CADSP database (https://smuonco.shinyapps.io/), we screened 288 drugs and identified antitumor drugs that were relatively sensitive to the DEGs.

### Sample collection

Tumor tissue and adjacent healthy tissue samples were collected from 22 patients with hepatocellular carcinoma at the Fifth Affiliated Hospital of Zhengzhou University from July 2023 to August 2023.

### Immunohistochemical (IHC) staining

Paraffin-embedded tissues were cut into 3 μm sections for IHC staining. The samples were incubated in a microwave oven at 100 °C in citrate buffer (pH 6.0) for 15 min and cooled naturally to room temperature for antigen recovery. After blocking with a mixture of methanol and 0.75% hydrogen peroxide, sections were incubated with anti-IGF1 primary antibody (D160510, Sangon Biotech, China; dilution 1:50), anti-CDKN2A primary antibody (D227441, Sangon Biotech, China; dilution 1:65), anti-BIRC5 primary antibody (D120314, Sangon Biotech, China; dilution 1:60) and anti-SPP1 antibody (D121078, Sangon Biotech, China; dilution 1:20) at 4 °C overnight. Then, the tissue sections were incubated with a secondary antibody conjugated to HRP (D110058, Sangon Biotech, China; dilution 1:500) and washed 3 times with TBST buffer. Finally, the sections were counterstained with hematoxylin, dehydrated and mounted.

### RNA isolation and real-time reverse transcription polymerase chain reaction (RT‒PCR)

Total RNA was extracted from the paraffin-embedded tumor and adjacent carcinoma tissues using an RNA kit (G3013, Wuhan Servicebio Technology Co., Ltd.). cDNA was amplified using primers specific for the reverse transcription kit (G3337, Wuhan Servicebio Technology Co., Ltd.). RT‒PCR was performed using a LightCycler 480 (Roche) under the following conditions: 95 °C for 30 s as the initial denaturation step, followed by 40 cycles of 95 °C for 15 s and 60 °C for 30 s, with a final melting curve from 65 to 95 °C in intervals of 0.5 °C to confirm the acquisition of a single fluorescence signal. GAPDH was used as a control. The RT‒PCR primer sequences (5’-3’) used were as follows:

IGF1—F GGTGGATGCTCTTCAGTTCGT; IGF1—R GCAATACATCTCCAGCCTCCTTA;

SPP1—F CAGTGATTTGCTTTTGCCTCC; SPP1- R ATCTGGGTATTTGTTGTAAAGCTGC;

BIRC5—F TTTGAGGAAACTGCGGAGAA; BIRC5—R TGATCTCCTTTCCTAAGACATTGC;

CDKN2A—F ATCGCGATGTCGCACGGTA; CDKN2A—R GGATGTCTGAGGGACCTTCC;

GAPDH F—GGAAGCTTGTCATCAATGGAAATC;

GAPDH R—TGATGACCCTTTTGGCTCCC.

### Statistical analysis

R software was used to perform all of the statistical analyses. The Wilcoxon rank-sum test and paired t test were used to evaluate differential expression levels between tumor and standard samples. P values less than 0.05 were considered to indicate statistical significance.

## Results

### Identification of DEGs related to autophagy

**Fig. 1:**
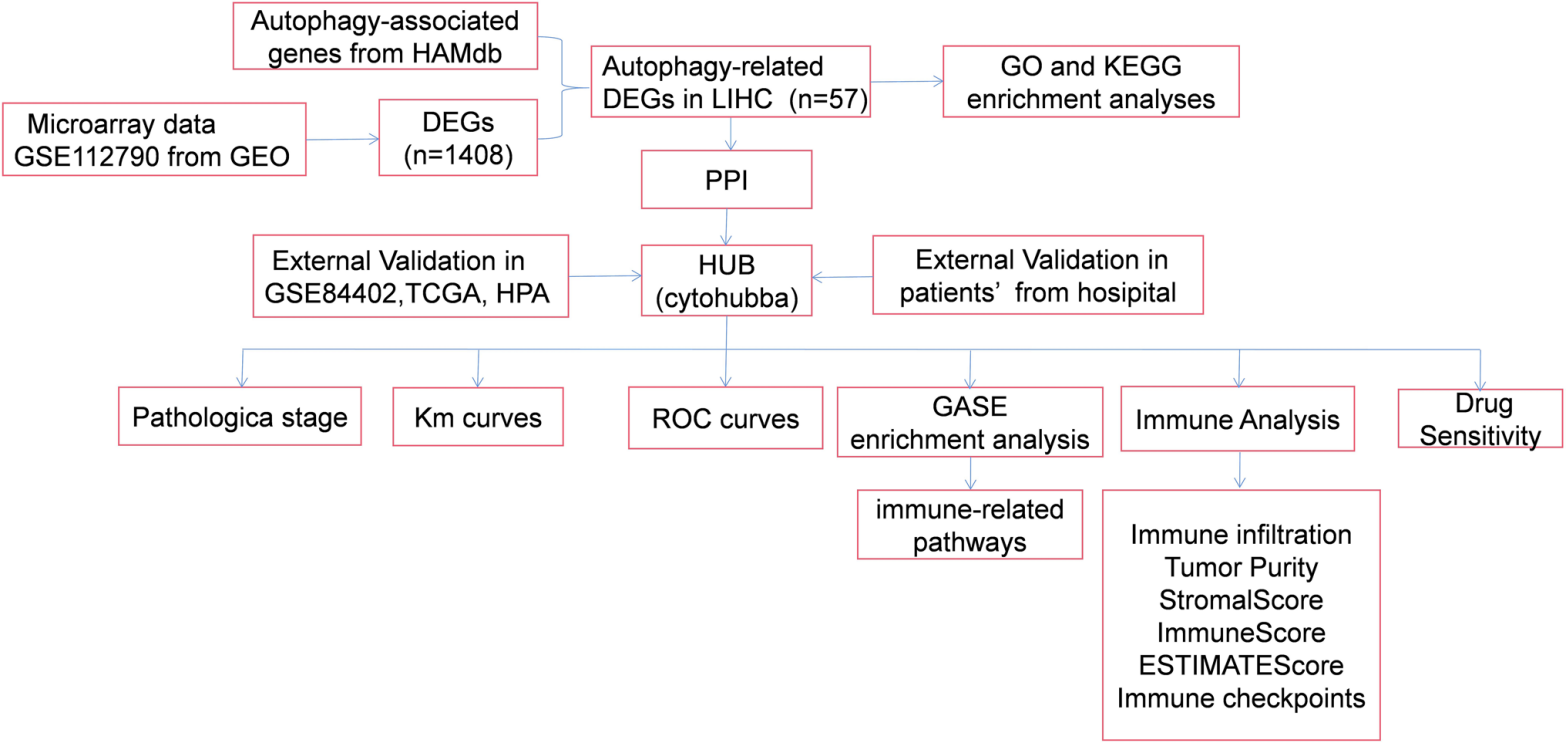
Flow chart of the study

**Fig. 2:**
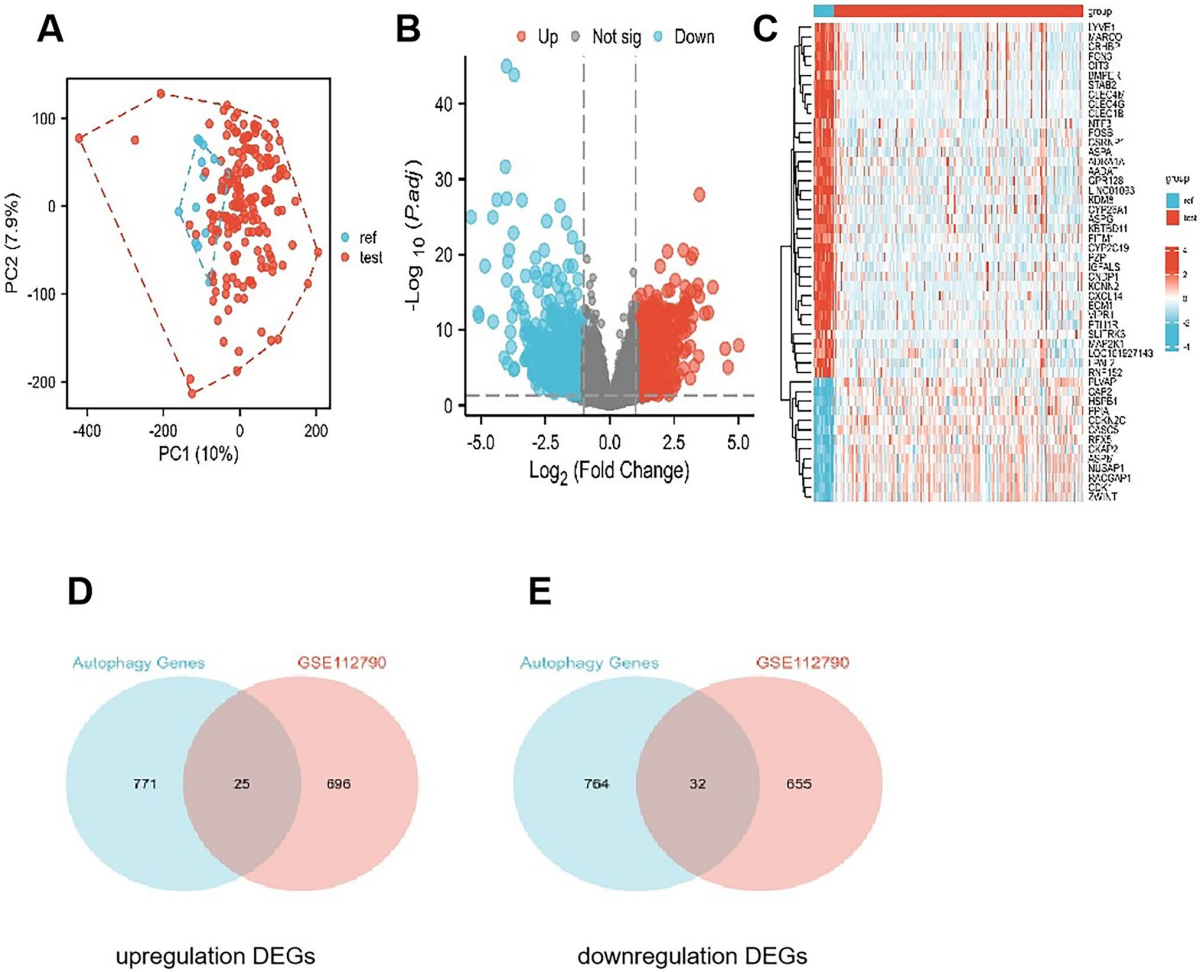
Identification of autophagy-related genes using GEO and TCGA datasets. **A** PCA array. **B** DEGs between the 183 HCC tumors and 15 adjacent liver samples. **C** Heatmap showing the expression levels of 57 autophagy-related DEGs in normal and tumor tissues. **D** Twenty-five overlapping genes identified as autophagy-related upregulated DEGs. **E** Thirty-two overlapping genes identified as autophagy-related downregulated DEGs

Our workflow is shown in Fig. 1. The GSE112790 dataset included 183 HCC tumors and 15 adjacent liver samples. The differentially expressed genes (DEGs) were screened with the following criteria: |logFC|> 1 and adj P < 0.05. As shown in Fig. 2A, these autophagy-related genes could distinguish between paracarcinoma tissue samples and HCC tissue samples, suggesting that autophagy-related genes play essential roles in the development of HCC. A total of 1408 differentially expressed mRNAs (DEMs) were identified between cancer tissue samples and paracarcinoma tissue samples in the dataset (Fig. 2B). Venn diagrams were drawn using an online Venn diagrams tool. There were 57 autophagy-related DEGs (Fig. 2C), including 25 upregulated DEGs (Fig. 2D, Table 1) and 32 downregulated DEGs (Fig. 2E, Table 1).

**Table 1:** Lists of DEGs upregulated/downregulated in dataset GSE112790

	DEGs	logFC	P.Value		DEGs	logFC	P.Value
Upregulated	ACLY	1.468191837	4.82671E-16	Downregulated	ACSL1	−1.075928641	0.000194901
	ATG10	1.072388841	2.07433E-08		AGTR1	−1.335235139	1.50655E-06
	AURKA	2.915479899	8.78278E-18		C9orf72	−1.261502629	1.85777E-08
	BIRC5	2.448049713	1.44369E-12		CCL2	−1.037983172	0.004781302
	CDKN2A	1.156043517	1.0346E-06		CD4	−1.290779059	1.35235E-09
	LAMP3	1.760371875	1.30157E-07		CXCL12	−2.886971734	3.79009E-09
	LAPTM4B	1.36569339	4.1246E-05		DCN	−2.412970763	4.9253E-06
	NPC1	1.004354241	3.23403E-07		FOXO1	−1.460038782	2.20456E-11
	NQO1	2.55721155	0.000236732		GABARAPL1	−1.595391176	3.60355E-09
	NUPR1	1.120569887	1.873E-05		HMOX1	−1.390442183	6.39839E-07
	OPTN	1.027072138	1.82287E-10		IGF1	−2.719606776	3.18948E-08
	PKM	1.333962652	8.98928E-05		IL33	−1.164820434	0.00041468
	PRKAA2	2.402493632	1.66195E-07		LEPR	−1.097147504	0.000290095
	PRKDC	1.018434064	4.05618E-06		MAP2K1	−1.440449481	2.41348E-22
	SCD	1.785828705	8.04476E-08		MYC	−1.215963778	0.001359702
	SMYD3	1.228144151	1.42898E-06		NAMPT	−1.578561822	3.60616E-08
	SPP1	3.127850195	2.44401E-05		NRBF2	−1.329540078	6.10759E-12
	SQSTM1	1.056034898	3.53294E-07		PDK4	−1.653121581	8.46198E-05
	TBC1D16	1.696896062	2.93205E-14		PLG	−1.44281363	0.001011795
	TBC1D7	1.321936925	1.00475E-13		RNF152	−1.446766729	2.28421E-26
	THBS2	1.367980564	0.003134061		S100A8	−2.608266562	1.04177E-07
	CAPN2	1.03729243	1.92284E-06		SRPX	−2.274284842	3.18912E-08
	HSP90AB1	1.021262117	3.17726E-11		VMP1	−1.147220421	0.000566033
	ITGA6	1.62202571	1.10828E-14		DIRAS3	−1.516147387	1.1634E-07
	MAPK9	1.006302098	2.76131E-08		DLC1	−1.142425427	2.87726E-07
					EDEM1	−1.08060739	2.70055E-14
					FOS	−3.303347669	7.35595E-15
					TUSC1	−1.277422155	0.000167065
					ALPL	−1.827097351	6.58066E-10
					CAPN3	−1.136690227	9.00929E-06
					NFKBIZ	−1.461020374	1.01395E-07
					RCAN1	−2.197725601	4.17554E-15

### Enrichment analysis of autophagy-related genes

In this study, we performed gene set enrichment analysis to observe the functional enrichment of autophagy-related DEGs in HCC (Table 1).

Gene Ontology (GO) enrichment analyses were conducted for the upregulated (Table 2) and downregulated DEGs related to autophagy (Table 3).

**Table 2:** GO-BP and KEGG pathway analyses of upregulated DEGs associated with autophagy

Ontology	Description	Pvalue	Count	GeneID
GO-BP	Regulation of cellular protein catabolic process	8.96E-07	6	AURKA/LAMP3/LAPTM4B/NUPR1/HSP90AB1/MAPK9
	Macroautophagy	2.41E-06	6	ATG10/NPC1/NUPR1/OPTN/PRKAA2/SQSTM1
	Peptidyl-serine phosphorylation	2.85E-06	6	AURKA/PRKAA2/PRKDC/SMYD3/HSP90AB1/MAPK9
	Peptidyl-serine modification	4.28E-06	6	AURKA/PRKAA2/PRKDC/SMYD3/HSP90AB1/MAPK9
	Regulation of proteasomal protein catabolic process	4.50E-06	5	AURKA/LAMP3/NUPR1/HSP90AB1/MAPK9
	Regulation of proteolysis involved in cellular protein catabolic process	9.82E-06	5	AURKA/LAMP3/NUPR1/HSP90AB1/MAPK9
	Regulation of protein catabolic process	1.04E-05	6	AURKA/LAMP3/LAPTM4B/NUPR1/HSP90AB1/MAPK9
	Negative regulation of cellular catabolic process	2.25E-05	5	LAMP3/LAPTM4B/NPC1/NUPR1/HSP90AB1
	Regulation of macroautophagy	5.29E-05	4	NPC1/NUPR1/OPTN/PRKAA2
	Negative regulation of catabolic process	5.89E-05	5	LAMP3/LAPTM4B/NPC1/NUPR1/HSP90AB1
KEGG	Fluid shear stress and atherosclerosis	4.36E-05	5	NQO1/PRKAA2/SQSTM1/HSP90AB1/MAPK9
	Focal adhesion	2.49E-04	5	SPP1/THBS2/CAPN2/ITGA6/MAPK9
	Autophagy—animal	6.92E-04	4	ATG10/PRKAA2/SQSTM1/MAPK9
	Necroptosis	1.08E-03	4	SQSTM1/CAPN2/HSP90AB1/MAPK9
	Mitophagy—animal	1.16E-03	3	OPTN/SQSTM1/MAPK9
	ECM-receptor interaction	2.07E-03	3	SPP1/THBS2/ITGA6
	Progesterone-mediated oocyte maturation	3.16E-03	3	AURKA/HSP90AB1/MAPK9
	PI3K-Akt signaling pathway	3.20E-03	5	PRKAA2/SPP1/THBS2/HSP90AB1/ITGA6
	Apoptosis—multiple species	3.89E-03	2	BIRC5/MAPK9
	Lysosome	6.52E-03	3	LAMP3/LAPTM4B/NPC1

**Table 3:** GO-BP enrichment and KEGG pathway analyses of downregulated DEGs associated with autophagy

Ontology	Description	Pvalue	Count	Gene ID
GO-BP	Regeneration	1.91E-08	7	CXCL12/HMOX1/IGF1/ MAP2K1/MYC/PLG/CAPN3
	Response to extracellular stimulus	7.89E-07	8	ACSL1/FOXO1/GABARAPL1/ HMOX1/PDK4/RNF152/FOS/ ALPL
	Cellular response to extracellular stimulus	2.58E-06	6	FOXO1/GABARAPL1/ HMOX1/PDK4/RNF152/FOS
	Positive regulation of autophagy	2.68E-06	5	C9orf72/DCN/FOXO1/ HMOX1/RNF152
	Positive regulation of nitric-oxide synthase biosynthetic process	2.71E-06	3	CCL2/IL33/NAMPT
	Nitric-oxide synthase biosynthetic process	5.29E-06	3	CCL2/IL33/NAMPT
	Regulation of nitric-oxide synthase biosynthetic process	5.29E-06	3	CCL2/IL33/NAMPT
	Epithelial cell proliferation	6.19E-06	7	AGTR1/CCL2/CXCL12/HMOX1/IGF1/MAP2K1/MYC
	Response to nutrient levels	6.47E-06	7	ACSL1/FOXO1/GABARAPL1/HMOX1/PDK4/RNF152/ALPL
	Positive regulation of cellular catabolic process	6.76E-06	7	C9orf72/DCN/FOXO1/HMOX1/IGF1/MYC/RNF152
KEGG	Autophagy—animal	8.50E-05	5	C9orf72/GABARAPL1/MAP2K1/NRBF2/VMP1
	Thyroid hormone signaling pathway	6.18E-04	4	FOXO1/MAP2K1/MYC/RCAN1
	FoxO signaling pathway	8.38E-04	4	FOXO1/GABARAPL1/IGF1/MAP2K1
	Yersinia infection	9.86E-04	4	CCL2/CD4/MAP2K1/FOS
	Breast cancer	1.28E-03	4	IGF1/MAP2K1/MYC/FOS
	Influenza A	2.23E-03	4	CCL2/IL33/MAP2K1/PLG
	Cytokine-cytokine receptor interaction	2.49E-03	5	CCL2/CD4/CXCL12/IL33/LEPR
	Colorectal cancer	2.75E-03	3	MAP2K1/MYC/FOS
	PD-L1 expression and PD-1 checkpoint pathway in cancer	3.03E-03	3	CD4/MAP2K1/FOS
	Rheumatoid arthritis	3.43E-03	3	CCL2/CXCL12/FOS

The top 10 pathways identified by the GO analyses are displayed in a bubble diagram.

**Fig. 3:**
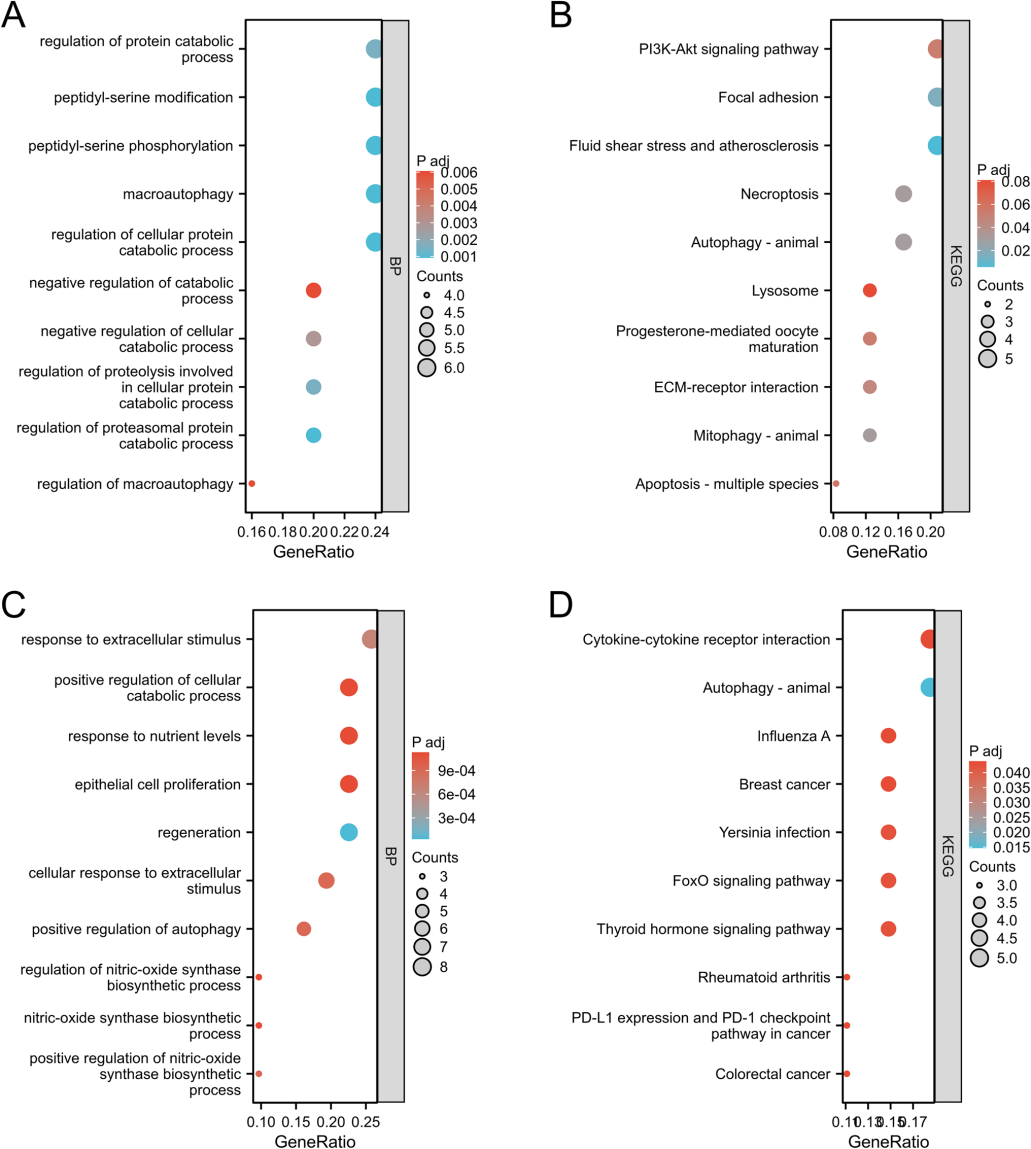
Enrichment analysis of autophagy-related genes by TCGA database. **A** GO-BP enrichment analysis of autophagy-related upregulated genes. **B** KEGG enrichment analysis of autophagy-related upregulated genes. **C** GO-BP enrichment analysis of autophagy-related downregulated genes. **D** KEGG enrichment analysis of autophagy-related downregulated genes

The coupregulated DEGs were mostly enriched in protein catabolic process, macroautophagy, peptidyl serine phosphorylation, peptidyl-serine modification and regulation of cellular protein catabolism process (Fig. 3A), and the codownregulated DEGs were mostly enriched in regeneration, response to extracellular stimuli, epithelial cell proliferation, response to nutrient levels, and positive regulation of cellular catabolic processes and regeneration (Fig. 3C).

Kyoto Encyclopedia of Genes and Genomes (KEGG) enrichment analyses were conducted for the upregulated (Table 2) and downregulated DEGs related to autophagy (Table 3).

The top 10 pathways identified by the KEGG analyses are displayed in a bubble diagram.

The coupregulated DEGs were mostly enriched in fluid shear stress and atherosclerosis, focal adhesion and the PI3K-Akt signaling pathway (Fig. 3B), and the codownregulated DEGs were mostly enriched in autophagy–animal and cytokine‒cytokine receptor interactions (Fig. 3D).

### Construction of a protein‒protein interaction (PPI) network via STRING and identification of HUB genes via Cytoscape software

**Fig. 4:**
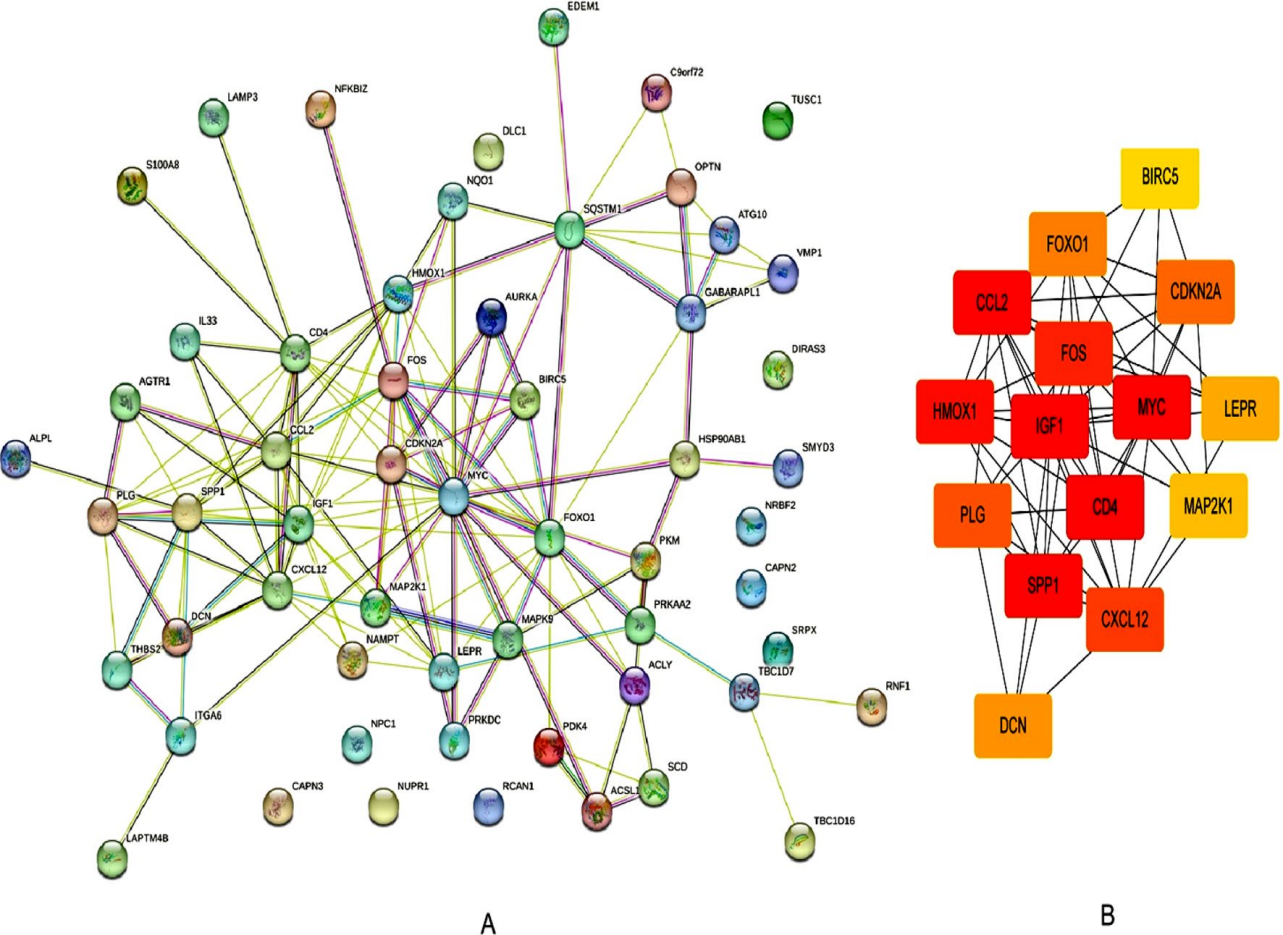
Identification of HUB genes by the STRING database. **A** PPI network of overlapping DEGs. **B** The most significant 15 node degree genes calculated by the cytoHubba app in Cytoscape. *IGF1, CDKN2A, BIRC5,* and *SPP1* were selected as the HUB genes. The node color intensities representing different genes correlate with the degree of expression values

STRING was used to construct a network for the DEGs associated with autophagy (Fig. 4A). After further analysis with Cytoscape, *IGF1, CDKN2A, BIRC5,* and *SPP1* were selected as the HUB genes and visualized (Fig. 4B). IGF1, CDKN2A, BIRC5, and SPP1 were selected as potential biomarkers (Table 4) and further validated in subsequent studies.

**Table 4:** Information on differentially regulated genes

Gene	Gene ID	Full name	Location	Functions of the encoded protein
*IGF1*	3479	Insulin-like growth factor 1	Secreted to blood	The protein encoded by this gene is similar to insulin in function and structure and is a member of a family of proteins involved in mediating growth and development. The encoded protein is processed from a precursor, bound by a specific receptor, and secreted
*CDKN2A*	1029	Cyclin-dependent kinase inhibitor 2A	Nucleoplasm and cytosol	Acts as a negative regulator of the proliferation of normal cells by interacting strongly with CDK4 and CDK6
*BIRC5*	332	Baculoviral IAP repeat-containing 5	Cytokinetic bridge	Multitasking protein that has dual roles in promoting cell proliferation and preventing apoptosis
*SPP1*	6696	Secreted phosphoprotein 1	Golgi apparatus, Secreted to blood	Major noncollagenous bone protein that binds tightly to hydroxyapatite. Appears to form an integral part of the mineralized matrix. Most likely important to cell–matrix interactions

### Database-based validation of the transcript levels of *IGF1*, *CDKN2A*, *BIRC5*, and *SPP1*

**Fig. 5:**
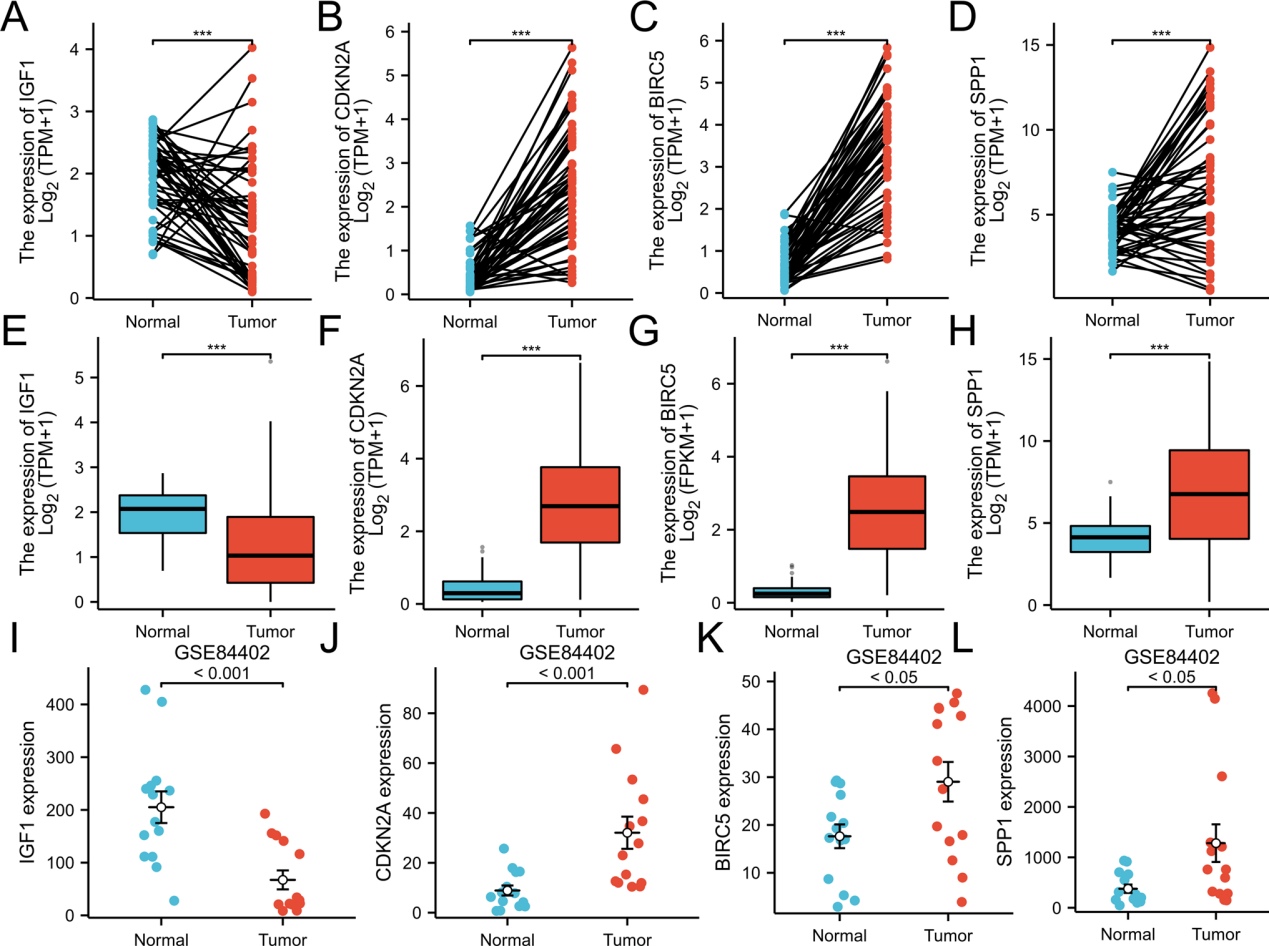
Expression levels of *IGF1, CDKN2A, BIRC 5,* and *SPP1* in HCC from the GEO and TCGA databases. **A**–**H** *IGF1, CDKN2A, BIRC5,* and *SPP1* mRNA expression levels are based on the TCGA database, including paired (**A**–**D**) and nonpaired samples (**E**–**H**). **I**–**L** The mRNA expression levels of *IGF1, CDKN2A, BIRC5,* and *SPP1* mRNA are based on GSE84402

**Fig. 6:**
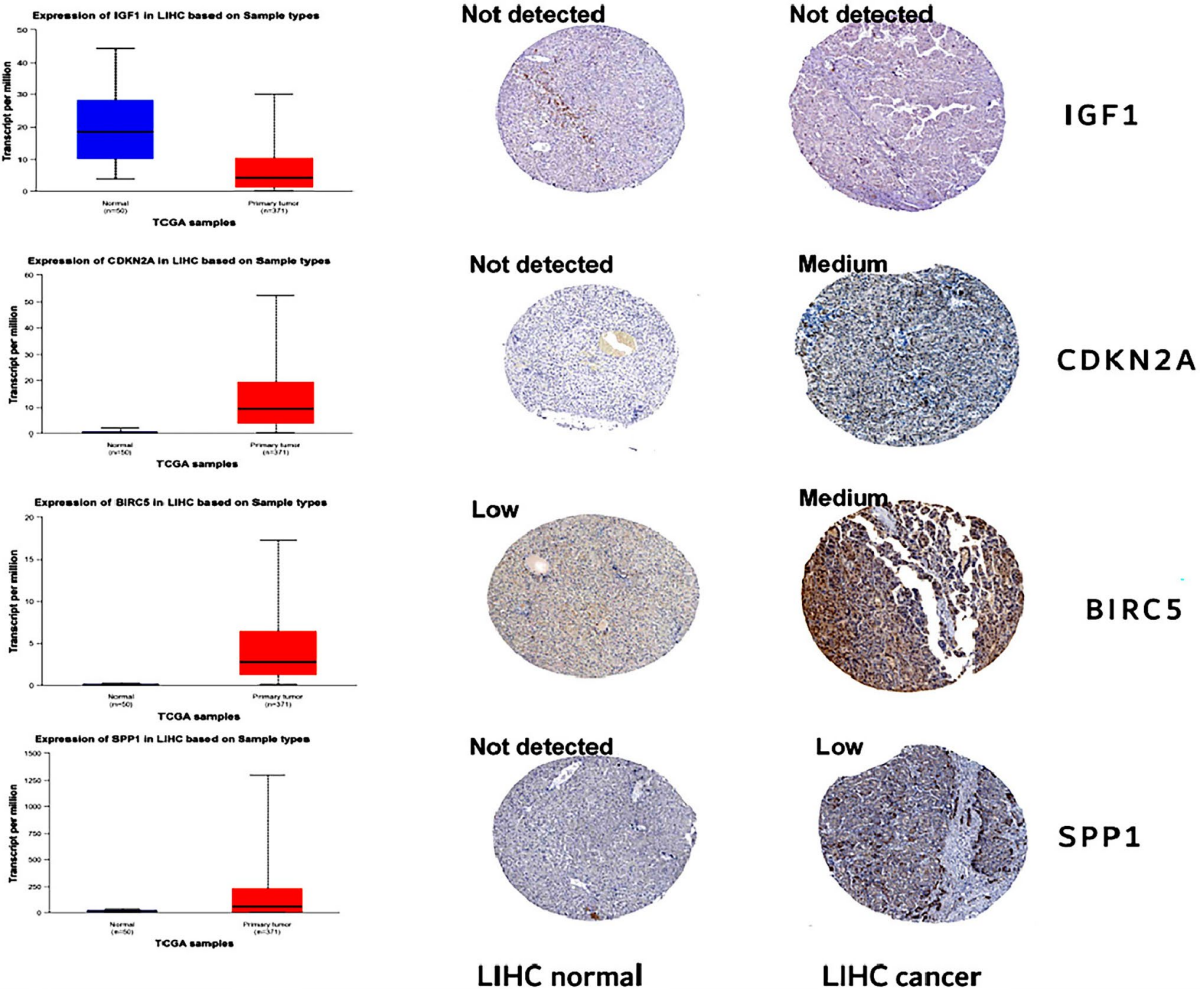
Levels of IGF1, CDKN2A, BIRC5, and SPP1 in normal individuals and patients with HCC from the HPA database

The expression levels of *IGF1, CDKN2A, BIRC5,* and *SPP1* mRNAs in paired (Fig. 5A–D) and nonpaired (Fig. 5E–H) samples in the TCGA database were evaluated. *CDKN2A, BIRC5,* and *SPP1* expression levels were significantly upregulated in tumor tissues (p < 0.001) (Fig. 5A–H), while *IGF1* expression was considerably downregulated (p < 0.001). We also used a GSE84402 dataset to validate the mRNA expression levels of *IGF1, CDKN2A, BIRC5,* and *SPP1* in hepatocellular carcinoma tissues and adjacent tissues. Compared with those in adjacent tissues, the expression levels of *CDKN2A, BIRC5,* and *SPP1* were significantly upregulated in tumor tissues (p < 0.05), while the expression of *IGF1* was considerably downregulated (p < 0.001) (Fig. 5I–L). Furthermore, immunohistochemical staining images from the HPA database indicated that the expression levels of *CDKN2A, BIRC5,* and *SPP1* were upregulated in tumor samples, while *IGF1* expression was not detected in either hepatocellular carcinoma tissues or adjacent tissues (Fig. 6). This finding was different from our previous analysis. We suspected that the author for the dataset did not choose an effective antibody or that the experimental conditions needed to be explored. These findings indicated that *CDKN2A, BIRC5,* and *SPP1* were upregulated in HCC, while *IGF1* expression was markedly downregulated.

### Diagnostic value of IGF1, CDKN2A, BIRC5 and SPP1 for HCC

**Fig. 7:**
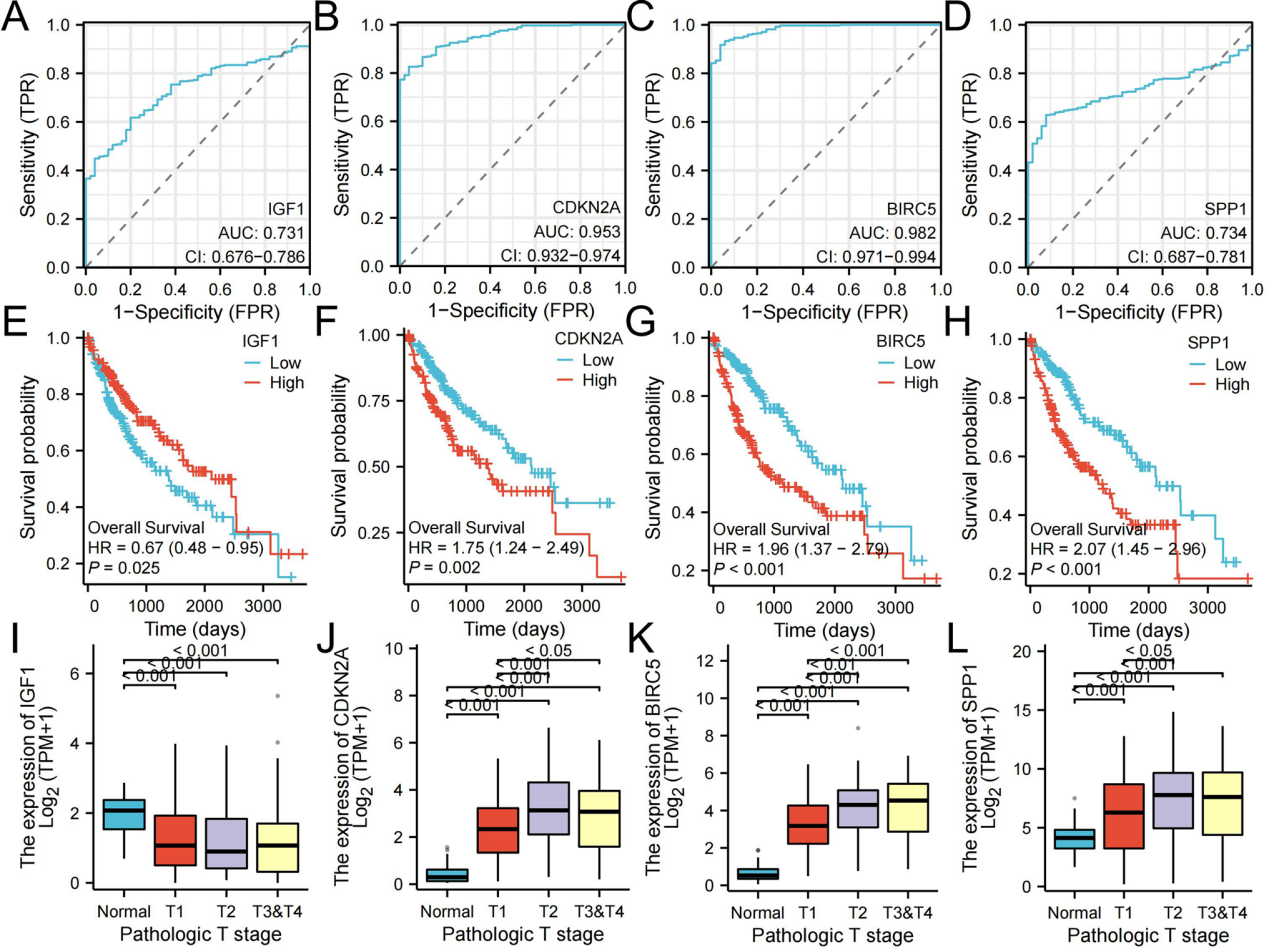
Analysis of the clinical implications of IGF1, CDKN2A, BIRC5, and SPP1 on the XIANTAO planform. **A**–**D** ROC curve analysis for IGF1, CDKN2A, BIRC5, and SPP1. **E**–**H** Overall survival analysis for IGF1, CDKN2A, BIRC5, and SPP1. **I**–**L** Analysis of the association of TNM stages with IGF1, CDKN2A, BIRC5, and SPP1

This study investigated the clinical diagnostic value of IGF1, CDKN2A, BIRC5, and SPP1 in HCC by constructing ROC curves. The areas under the curve (AUCs) were 0.731, 0.953, 0.982, and 0.734, indicating the diagnostic value of IGF1, CDKN2A, BIRC5, and SPP1 for HCC, respectively (Fig. 7A–D).

### Overall survival analysis for patients with HCC stratified by IGF1, CDKN2A, BIRC5 and SPP1 levels

The overall survival of patients with HCC stratified by IGF1, CDKN2A, BIRC5, and SPP1 levels was analyzed by Kaplan‒Meier plotter to further investigate whether IGF1, CDKN2A, BIRC5, or SPP1 had an effect on overall survival. Our findings suggested that high levels of CDKN2A, BIRC5, and SPP1 were associated with poor overall survival in patients with HCC, suggesting that BIRC5, CDKN2A, and SPP1 are associated with HCC progression and could be used as tumor biomarkers in patients with HCC. IGF1 was at lower levels in tumor tissues than in adjacent tissues, possibly because of its association with lower survival (Fig. 7E–H).

### Analysis of the associations of tumor node metastasis (TNM) stages with IGF1, CDKN2A, BIRC5 and SPP1 levels

We analyzed the associations of TNM stage with IGF1, CDKN2A, BIRC5, and SPP1. We found significant differences in different T stages compared to regular level, suggesting that HUB genes may be related to the progression of HCC (Fig. 7I–L).

### GSEA revealed pathways associated with IGF1, CDKN2A, BIRC5 and SPP1 expression in HCC

**Fig. 8:**
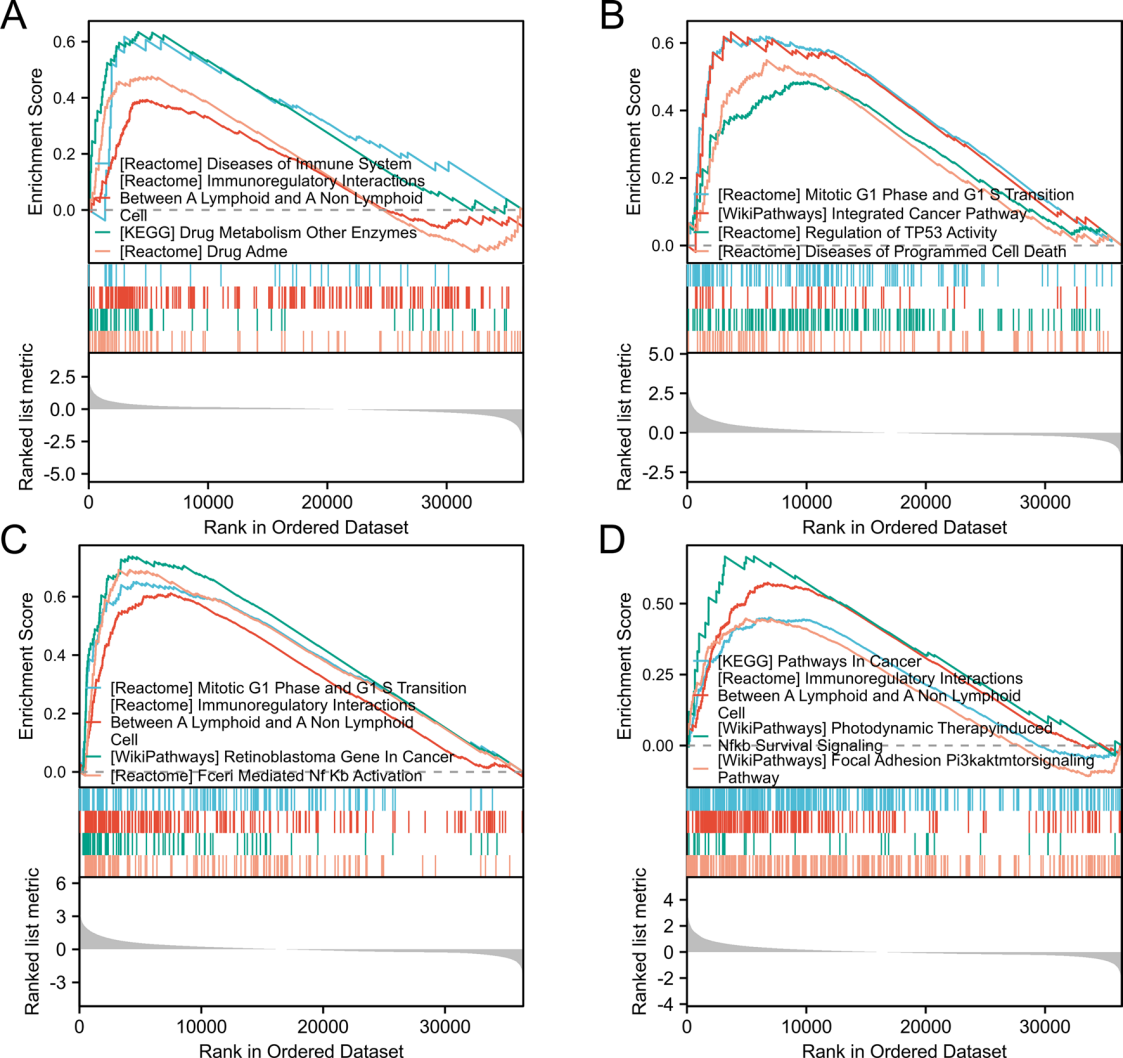
GSEA for IGF1, CDKN2A, BIRC5, and SPP1 by the TCGA database

GSEA indicated that IGF1-related genes were mainly enriched in immune-related pathways, such as disease of the immune system, immunoregulatory interactions between lymphoid and nonlymphoid cells, drug metabolism, other enzymes, and drug ADME (Fig. 8A). CDKN2A-related genes were mainly enriched in the mitotic G1 phase and G1 transition, integrated cancer pathway, regulation of TP53 activity, and disease of programmed cell death (Fig. 8B). BIRC5-related genes were mainly enriched in the mitotic G1 phase and G1 transition, immunoregulatory interaction, lymphoid and nonlymphoid cells, retinoblastoma gene in cancer, and FceRI-mediated NFkb activation (Fig. 8C). SPP1-related genes were mainly enriched in pathways related to cancer, immunoregulatory interactions between lymphoid and nonlymphoid cells, photodynamic therapy-induced NFkb survival signaling, focal adhesion PI3K, AKT, and the mTOR signaling pathway (Fig. 8D).

### Correlation analysis of IGF1, CDKN2A, BIRC5 and SPP1 with the TME in HCC

**Fig. 9:**
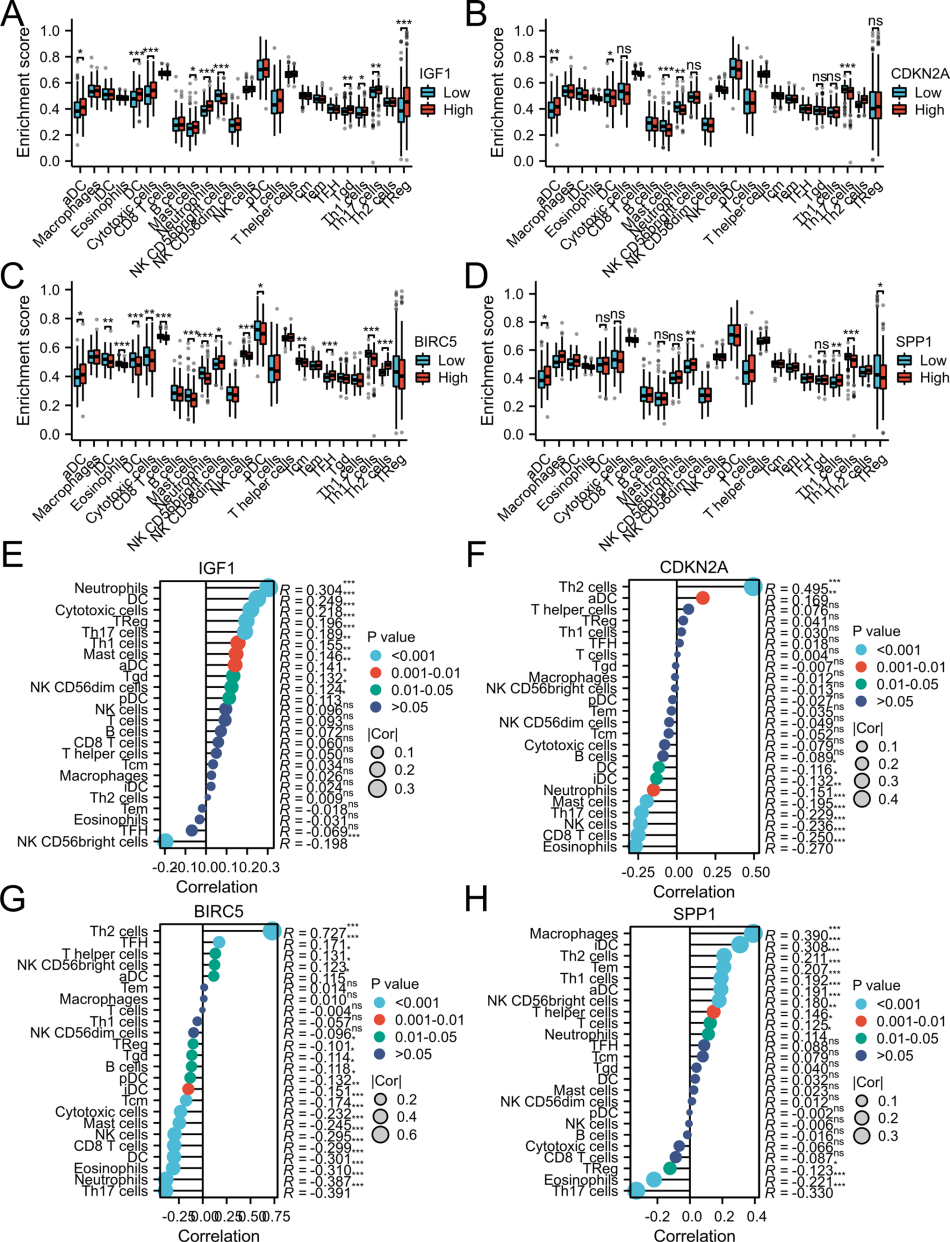
Levels of IGF1, CDKN2A, BIRC5, SPP1 and their correlations with the TME on the XIANTAO planform. **A**–**D** Immune infiltration analysis for IGF1, CDKN2A, BIRC5 and SPP1. **E**–**H** Correlation levels of IGF1, CDKN2A, BIRC5, and SPP1 with the level of immune infiltration

**Fig. 10:**
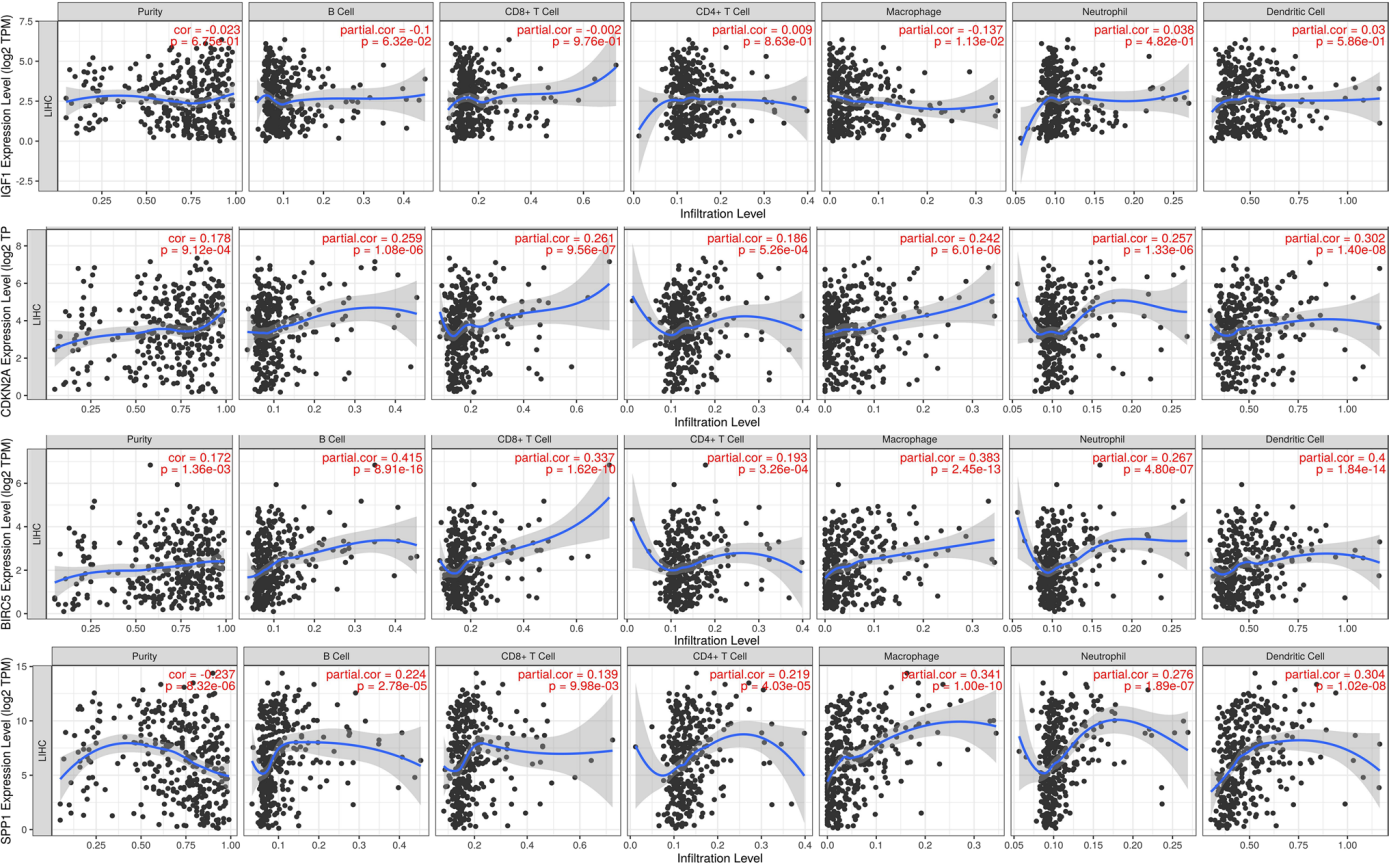
Immune infiltration analysis of IGF1, CDKN2A, BIRC5, and SPP1 performed using the TIMER database

**Fig. 11:**
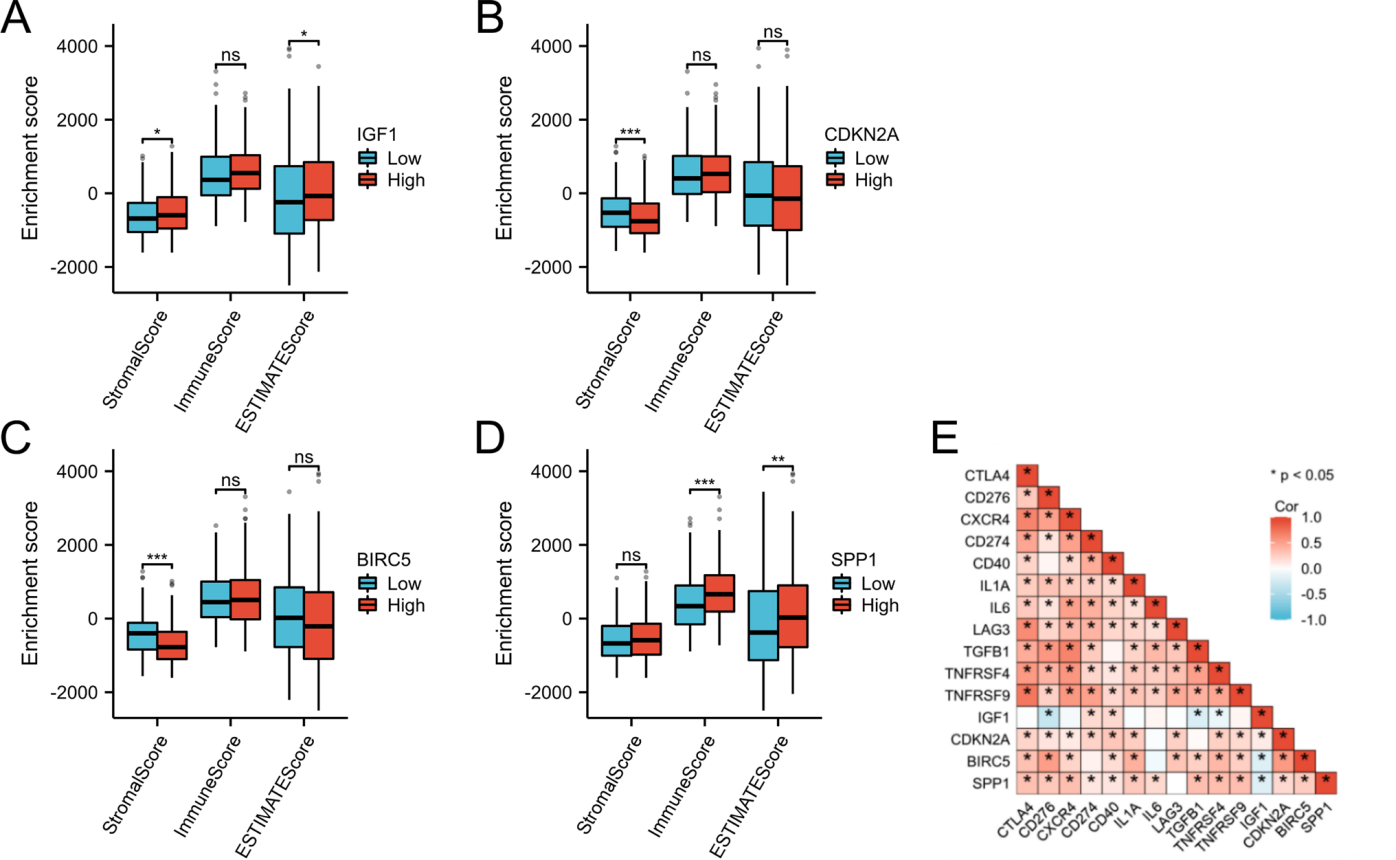
Immune analysis of IGF1, CDKN2A, BIRC5, and SPP1 by the TCGA database. **A**–**D** Correlations of the estimated proportions of immune and stromal cells with IGF1, CDKN2A, BIRC5, and SPP1 levels in HCC. **E** Various immune checkpoints were associated with IGF1, CDKN2A, BIRC5, and SPP1

As demonstrated in Fig. 9A, patients with lower level of IGF1 had lower numbers of memory dendritic cells (DCs), aDCs, cytotoxic cells, mast cells, neutrophils, Tgd, Th1 cells, Th17 cells, and Tregs. Additionally, as depicted in Fig. 9B–D, patients in the groups with high CDKN2A, BIRC5, and SPP1 had high Th2 cells and low Th 17 cells and eosinophils. According to Fig. 9E, patients with high IGF1 had negative correlations with NK and CD56bright cells but positive correlations with neutrophils, DCs, cytotoxic cells, Tregs, Th1 cells, Th17 cells, Mast cells, Tgd and NK CD56dim cells. As demonstrated in Fig. 9F–H, patients with high CDKN2A, BIRC5 and SPP1 had negative correlations with Th17 cells and eosinophils but positive correlations with Th2 cells. Neutrophils, Th17 cells, and eosinophils may be the most prevalent differential immune cells. Th2 cells may play a role in promoting cancer. In this research, we further assessed the correlations between immune infiltration levels and the expression levels of IGF1, CDKN2A, BIRC5, and SPP1 in HCC via the TIMER database. As shown in Fig. 10, IGF1 was negatively correlated with the infiltration of macrophages (p = 1.13 × 10 − 2); moreover, CDKN2A, BIRC5, and SPP1 levels were positively correlated with the infiltration of macrophages, B cells, CD4 + T cells, CD8 + T cells, dendritic cells, and neutrophils. Figure 11A–D shows that there was variability in the stromal, immune, and estimated scores of patients with HCC, indicating some differences in the TME according to the level of IGF1, CDKN2A, BIRC5, and SPP1. Various immune checkpoints were associated with IGF1, CDKN2A, BIRC5, and SPP1 (Fig. 11E).

### Possible sensitivity to therapeutic drugs

**Fig. 12:**
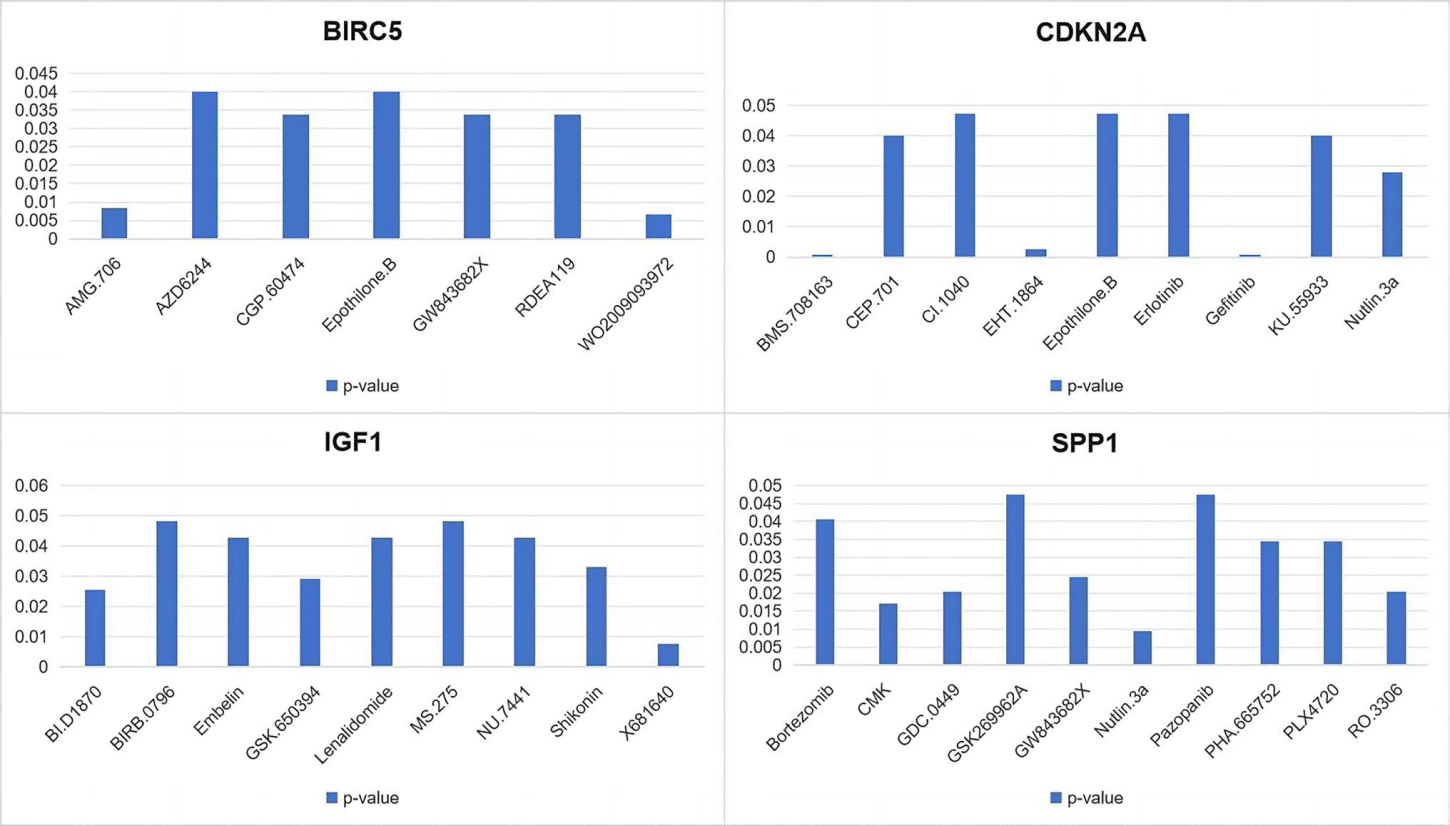
Possible Sensitivity to Therapeutic Drugs according to the CADSP database

Using the CADSP database, we screened 288 drugs and identified antitumor drugs that were relatively sensitive to DEGs. Patients with high level of CDKN2A and BIRC5 had the lowest half maximal inhibitory concentrations (IC50) for Epothilone B, while SPP1 was inhibited by bortezomib. Shikonin (SHK) had the lowest IC50 in patients in the group with high IGF1 level. We identified 35 tumor-sensitive drugs targeting the HUB genes (Fig. 12).

### External validation in patients with HCC from the hospital

**Fig. 13:**
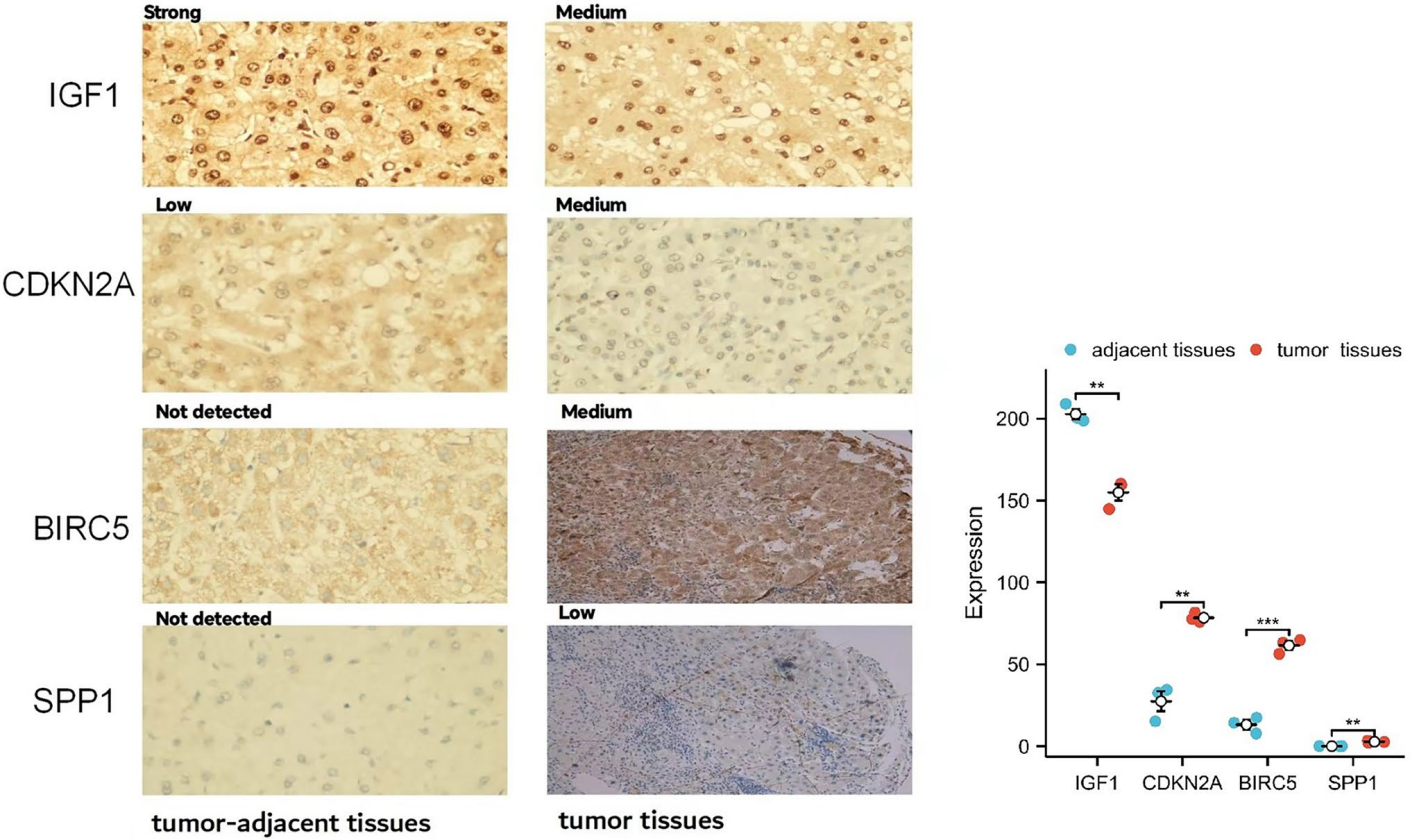
Levels of IGF1, CDKN2A, BIRC5, and SPP1 in patient tissues by IHC. Levels of CDKN2A, BIRC5, and SPP1 in HCC were increased compared with tumor-adjacent tissues. However, IGF1 expression was downregulated in HCC tissues compared with adjacent tissues

We validated the protein levels of IGF1, CDKN2A, BIRC5, and SPP1 in HCC by IHC (Fig. 13). CDKN2A, BIRC5, and SPP1 were upregulated in HCC tissues by IHC. However, IGF1 was downregulated in HCC tissues compared with adjacent tissues.

**Fig. 14:**
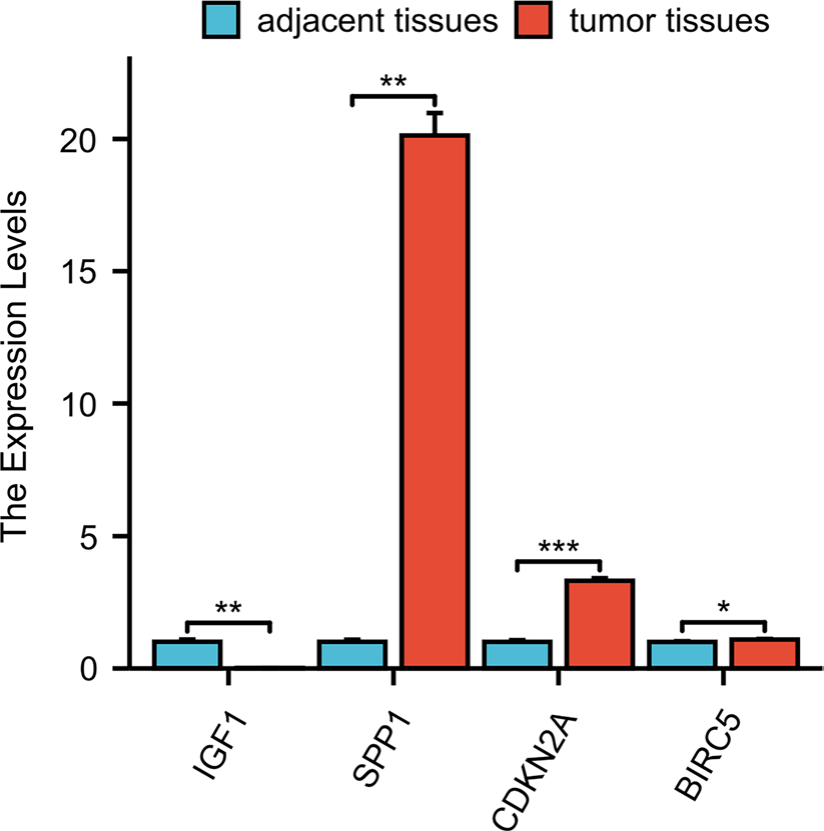
Expressions of *IGF1, CDKN2A, BIRC5,* and *SPP1* in patient tissues by RT‒PCR. The expressions of *CDKN2A, SPP1,* and *BIRC5* in HCC were increased compared with tumor-adjacent tissues. However, *IGF1* expression was downregulated in HCC tissues compared with that in adjacent tissues

We also validated the expressions of *IGF1, CDKN2A, BIRC5,* and *SPP1* in HCC by RT‒PCR (Fig. 14). *CDKN2A, SPP1,* and *BIRC5* were upregulated in HCC tissues according to RT‒PCR; *IGF1* was downregulated in HCC tissues compared with adjacent tissues.

## Discussion

In this study, we investigated autophagy-related genes and evaluated immune cell infiltration in HCC via bioinformatics methods, which could provide new perspectives for immunotherapy. We first collected the GSE112790 dataset, which included 183 HCC tumor samples and 15 adjacent liver samples. Then, 57 autophagy-related genes were identified between the tumor and adjacent tissue samples. Subsequently, we constructed a PPI network and identified four HUB genes, namely, *IGF1, CDKN2A, BIRC5,* and *SPP1*. The results of these analyses were confirmed by IHC and RT‒PCR. The protein of CDKN2A, BIRC5, and SPP1 were upregulated in HCC tissues by IHC. However, IGF1 was downregulated in HCC tissues compared with adjacent tissues. The mRNA of *CDKN2A, SPP1,* and *BIRC5* were upregulated in HCC tissues according to RT‒PCR; the mRNA of *IGF1* was downregulated in HCC tissues compared with adjacent tissues.

IGF1 is a RAS pathway-related protein that is present in plasma in all tissues [8]. The protein encoded by this gene is similar to insulin in function and structure and is a member of a family of proteins involved in mediating growth and the development of multiple diseases [9]. Our study revealed that IGF1, an autophagy-related protein, is expressed at lower levels in HCC tissues than in para-adjacent tissues and may be involved in the prognostic regulation of HCC.

The IGF1 level was greater in adjacent nonneoplastic liver tissues than in tumor tissues, which was correlated with significantly worse survival after HCC resection [10]. Multivariate analysis revealed that higher IGF-1 in adjacent nonneoplastic liver tissue remained an independent predictor of poor outcome [10]. Activation of the IGF1 signaling cascade leads to upregulation of downstream mitogens, including MAPK and PI3K/Akt. During liver carcinogenesis, the secretion of IGF1 by adjacent hepatocytes may lead to paracrine stimulation of HCC and more aggressive tumor behavior [11]. Increased levels of insulin-like growth factor 1 are associated with cancer development, and circulating IGF1 is positively associated with breast cancer risk [12]. The serum IGF1 concentration is greater in patients with melanoma than in healthy individuals [13]. Notably, elevated IGF1 is closely associated with drug resistance. After gefitinib treatment, liver cells exhibit increased phosphorylation of IGF1R and AKT in HCC, indicating that activation of IGF1R signaling may contribute to gefitinib resistance [14]. Suppressor of IGF1 signaling plays essential roles in cancer therapy. Therapeutic strategies, including IGF1 and IGF1R antibodies and tyrosine kinase inhibitors, have been developed, and both show good responses [15]. Targeting IGF1-associated lncRNAs has potential in cancer therapy. For example, silencing lncNEAT1 inhibited chemoresistance in gastric cancer [16]. These findings indicate that IGF1 plays a specific role in the development of cancer. Most importantly, elevated IGF1 is strongly associated with resistance to various chemotherapies, which may be modulated by IGF1-associated lncRNAs [17]. Finally, targeting IGF1-associated lncRNAs is also expected to prevent and reverse chemoresistance. More precise in vivo and in vitro experiments are needed to verify this mechanism in the future.

In the present study, GSEA indicated that IGF1-related genes were mainly enriched in immune-related pathways, such as disease of the immune system, immunoregulatory interactions between lymphoid and nonlymphoid cells, drug metabolism, other enzymes, and drug ADME. Therefore, we hypothesized that regulating IGF1, CDKN2A, BIRC5, and SPP1 levels may affect the tumor immune microenvironment in HCC. Previous reports have demonstrated a close relationship between immunity and autophagy [18]. Recent studies have shown that autophagy can direct the immune response by regulating the secretion of cytokines and immune cells [19]. The tumor microenvironment (TME) mainly comprises tumor cells and their surrounding immune and inflammatory cells, tumor-associated fibroblasts and nearby stromal tissues, microvessels, various cytokines, and chemokines. The TME can be divided into an immune microenvironment dominated by immune cells and a nonimmune microenvironment dominated by fibroblasts. We further assessed the correlations between immune infiltration levels and IGF1, CDKN2A, BIRC5, and SPP1 levels in HCC via the TIMER database. The results showed that IGF1, CDKN2A, BIRC5, and SPP1 levels correlated with infiltrating macrophage levels (Fig. 10). In the tumor–immune microenvironment, tumor-associated macrophages (TAMs) are recruited and activated by different chemokines and differentiate into proinflammatory and antitumor-active M1-TAMs and into anti-inflammatory and tumor-promoting M2-TAMs. The two forms of polarization are interconverted in the presence of certain stimuli [20]. TAMs can induce autophagy in HCC cells and attenuate the toxic effects of oxaliplatin. This autophagy-mediated drug resistance mechanism provides a novel therapeutic strategy [21]. Regulating autophagy in TAMs may be a promising strategy for inhibiting HCC progression. Modulated by genetic engineering techniques, macrophages can target specific antigens, such as CD19, CD22, and Her2, to identify tumor cells [22]. Chimeric antigen receptor macrophage (CAR-M) cells can phagocytose tumor cells, secrete proinflammatory cytokines to alter the tumor immune microenvironment, and present tumor antigens to T cells to activate the immune response [23]. CAR-M technology is constantly evolving; however, due to the heterogeneity of HCC, it remains challenging to explore specific targets in liver tumor cells and to engineer macrophages in the liver TME.

We also found significant associations of CD276, CD40, and TNFRSF4 with IGF1, CDKN2A, BIRC5 and SPP1. Targeting these immune checkpoints, such as inhibitors that target checkpoint molecules, may enhance the body's immune function. Combining ICB with other treatments may improve the immunological conditions of the TME, thus enhancing the antitumor immune response. Immune checkpoint blockade (ICB) has been successful in treating more immunogenic tumors [24]. However, ICB is still largely ineffective in patients with tumors that are not infiltrated by immune cells (cold tumors). ICB can be combined with compounds capable of converting noninflammatory tumors into inflammatory tumors to further increase the number of appropriate tumor types. This may, in turn, increase the sensitivity of these tumors to ICB therapy.

Finally, by using the CADSP database, we screened 288 drugs and identified antitumor drugs that are relatively sensitive to IGF1, CDKN2A, BIRC5, and SPP1. Epothilone B had the lowest IC50 for CDKN2A and BIRC5, while bortezomib had the lowest IC50 for SPP1. The patients in the group with high levels of IGF1 had the lowest IC50 for SHK; thus, these patients are more sensitive to SHK. Although the effect of chemotherapy on liver cancer is limited, it can improve the prognosis of specific patients and can extend their lives to a certain extent. SHK is a natural molecule isolated from peramina that shows great potential in anticancer therapy. SHK can also modulate the immunosuppressive TME by inhibiting tumor cell glycolysis and repolarizing tumor-associated macrophages [25]. M2-TAMs exert anti-inflammatory and tumorigenic effects. SHK can reduce the production of lactate acid, an essential driver of TAM2 polarization, thus repolarizing M2-TAMs [26]. PD-L1 is the primary ligand for PD-1. PD-L1 expression is an immune evasion mechanism in various malignancies [27]. SHK promotes PD-L1 degradation and suppresses the immune escape of pancreatic cancer cells by inhibiting the NF-κB/STAT3 and NF-κB/CSN5 signaling pathways [28]. Autophagy is a form of programmed cell death; therefore, the regulation of autophagy can be an effective intervention strategy for cancer therapy. Enhanced autophagy is accompanied by RIPK1- and RIPK3-dependent necrotizing apoptosis induced by SHK stimulation [29]. However, SHK is a hydrophobic natural molecule with unsatisfactory solubility and rapid intestinal absorption, resulting in low oral bioavailability [30]. Given the shortcomings of SHK, significant efforts have been made to develop various nanodrug delivery systems. A mannosylated lactoferrin nanosystem (Man-LF NPs) was prepared to deliver SHK and JQ1, which target colon cancer cells and TAMs [31]. When combined with other immunodrugs, JQ1 effectively reduced PD-L1 expression in tumor cells [32].

In this study, we screened and validated four autophagy-related genes associated with immune infiltration and prognosis in patients with HCC. It provided new insights into the HCC immune microenvironment and provide new perspectives on autophagy gene-targeted immunotherapy.

A limitation of this study is that our data are mainly derived from public data, which is available in limited amounts; moreover, the clinical validation samples were limited. Trials with sufficiently large sample sizes are needed for validation in the future.

## Conclusions

We identified and validated autophagy genes associated with HCC and revealed that HUB genes were correlated with the TME in HCC. These findings can provide new perspectives on autophagy gene-targeted immunotherapy.

## Data Availability

GEO database (https://www.ncbi.nlm.nih.gov/); TCGA database( https://portal.gdc.cancer.gov); HAMdb (http://hamdb.scbdd.com/); Bioinformatics online tool (http://www.bioinformatics.com.cn/); The String database (www.string-db.org); the HPA database (http://www.proteinatlas.org/); The TIMER database (https://cistrome.shinyapps.io/timer/); the XIANTAO planform (https://www.xiantaozi.com/); the CADSP database (https://smuonco.shinyapps.io/).
